# The emergence of sex differences in PTSD symptoms across development: evidence from the ALSPAC cohort

**DOI:** 10.1017/S0033291719001971

**Published:** 2020-07

**Authors:** Katharina Haag, Abigail Fraser, Rachel Hiller, Soraya Seedat, Annie Zimmerman, Sarah L. Halligan

**Affiliations:** 1 Department of Psychology, University of Bath, Bath, UK; 2 Population Health Sciences, Bristol Medical School, University of Bristol, Bristol, UK; 3 Medical Research Council (MRC) Integrative Epidemiology Unit at the University of Bristol, Bristol, UK; 4 Department of Psychiatry, Medical Research Council (MRC) Unit on Anxiety and Stress Disorders, Stellenbosch University, Tygerberg, Western Cape, South Africa; 5 School of Psychological Science, University of Bristol, Bristol, UK; 6 Department of Psychiatry and Mental Health, University of Cape Town, Cape Town, South Africa

**Keywords:** ALPSAC, childhood/adolescence, longitudinal, PTSD, sex-differences

## Abstract

**Background:**

Cross-sectional evidence suggests females in late adolescence exhibit higher rates of post-traumatic stress symptoms (PTSS) than males and younger age groups. However, longitudinal evidence is limited, and underlying factors are not well understood. We investigated the emergence of sex differences in PTSS from childhood to adolescence in a large, longitudinal UK cohort, and tested whether these could be explained by overlap between PTSS and depressive symptoms, or onset of puberty.

**Methods:**

Trauma exposure and PTSS were assessed at ages 8, 10, 13 (parent-report) and 15 (self-report) years in a sub-sample of 9966 children and adolescents from the ALSPAC cohort-study. Analyses of PTSS focused on those who reported potential trauma-exposure at each time-point (ranged from *n* = 654 at 15 years to *n* = 1231 at 10 years). Age at peak-height velocity (APHV) was used as an indicator of pubertal timing.

**Results:**

There was no evidence of sex differences in PTSS at ages 8 and 10, but females were more likely to show PTSS at ages 13 (OR 1.54, *p* = 0.002) and 15 (OR 2.04, *p* = .001), even once symptoms related to depression were excluded. We found little evidence that the emergence of sex differences was related to pubertal timing (as indexed by APHV).

**Conclusions:**

Results indicate that females show higher levels of PTSS in adolescence but not during childhood. The emergence of this sex difference does not seem to be explained by overlap with depressive symptoms, while the influence of pubertal status requires further investigation.

## Introduction

Post-traumatic stress disorder (PTSD) is a potentially debilitating condition, characterized by persistent symptoms of intrusive re-experiencing, avoidance of trauma-related stimuli, alterations in mood and cognitions, and heightened arousal and reactivity. In adults, higher rates of PTSD in females than in males have been consistently observed, with the estimated lifetime-prevalence being twice as high for females (Kessler *et al*., 1995). Females also tend to exhibit greater overall posttraumatic stress symptom (PTSS) severity (Tolin and Foa, 2006). It has been hypothesised that the higher prevalence of PTSD in women may be a consequence of sex differences in neuroendocrine, hormonal and stress response systems, types of trauma experienced, coping strategies, and socialization (for reviews, see Olff *et al*., 2007, Garza and Jovanovic, 2017). Meta-analytic evidence based on cross-sectional data suggests that the relative risk of developing PTSS for females *v.* males increases with age at trauma exposure up until adolescence (Trickey *et al*., 2012). However, longitudinal study is required to gather more precise information about when such sex differences emerge during development.

Observations of age-related effects on sex differences in PTSS prevalence concord with findings for major depression, where higher rates are reported in females *v.* males from adolescence onwards (Wade *et al*., 2002). Evidence indicates that pubertal stage, rather than chronological age, may be a more reliable predictor of the latter effect (Angold and Costello, 2006). In the PTSD field, it has similarly been hypothesised that hormonal changes associated with the onset of puberty (e.g. in adrenal androgens and sex steroids) could play a role in heightening female vulnerability to the effects of trauma, likely by influencing both general physiological systems such as neurotransmitter or stress systems, as well by interacting with the other sex specific vulnerability factors (Garza and Jovanovic, 2017). However, associations between pubertal timing and PTSS vulnerability have yet to be demonstrated.

In the current study, we conducted an analysis of sex differences in the prevalence of PTSS symptoms in a large longitudinal UK birth cohort. The longitudinal design included symptom assessments at 8, 10, 13 and 15 years, which allowed us to study the emergence of sex differences in PTSS from late childhood to adolescence within the same cohort. We also conducted a preliminary examination of potential contributors to sex differences. Specifically, we explored: (a) whether pubertal status contributes to the hypothesised emergence of sex differences in PTSD in adolescence and (b) whether the overlap with depressive symptoms would explain PTSD effects.

## Methods

### The Avon Longitudinal Study of Parents and Children (ALSPAC)

The Avon Longitudinal Study of Parents and Children (ALSPAC) is a prospective birth cohort study, which recruited 14 541 pregnant women living in the geographic area of Avon, UK, with expected delivery dates between 1 April 1991 and 31 December 1992. Ethical approval for the study was obtained from Local Research Ethics Committees, and the ALSPAC Ethics and Law Committee. The authors assert that all procedures contributing to this work comply with the ethical standards of the relevant national and institutional committees on human experimentation and with the Helsinki Declaration of 1975, as revised in 2008. With additional recruitment happening later, the enrolled sample for whom at least one assessment was completed consists of 15 589 children, resulting from 15 454 pregnancies. Data from the parents and these children have been collected on a regular basis since the child was born (Boyd *et al*., 2013; Fraser *et al*., 2013), and the ALSPAC study website provides a complete data dictionary and variable search tool (http://www.bristol.ac.uk/alspac/researchers/our-data/). The current study focuses on assessments completed during research clinics at 8, 10, 13 and 15 years. Informed consent for the use of data collected via questionnaires and clinics was obtained from participants following the recommendations of the ALSPAC Ethics and Law Committee at the time.

### Materials

#### Development and Well-Being Assessment (DAWBA)

The DAWBA provides a measure of DSM- and ICD-based diagnoses of all major psychiatric disorders for the ages 5–16, with established validity and reliability (Goodman *et al*., 2000). In ALSPAC, the DAWBA PTSD assessment was completed by the child's current carer (predominantly the biological mother), at clinic assessments at the ages 8, 10 and 13, and by the adolescents themselves at the age of 15. Participants were first given the screening question – ‘*During your* (*child*'*s*) *lifetime, has anything exceptionally stressful happened to you* (*them*) *that would really upset almost anyone, such as being involved in a terrible accident, or being abused, or some other sort of disaster?*’. If this was answered affirmatively, the carer/adolescent was asked to provide details on the age at which the event had taken place, and whether the event had caused significant distress at exposure (1 = ‘*yes*’, 2 = ‘*no*’). Carers also rated associated past month PTSS in their child/adolescent at age 8, 10 or 13 years (13 items), and adolescents self-rated their PTSS at 15 years (15 items, due to two symptoms being added to the latest DAWBA version), on a scale of 0 = *‘no’*, 1 = *‘a little’*, or 2 = *‘a lot’*. Responses were summed to create symptom scores of 0–26 and 0–30 points respectively. For the current analyses, we utilised two variables to capture PTSD symptom experiences: (a) the total symptoms score; (b) a binary variable indicating the presence PTSD symptoms, defined as at least one PTSD symptom being present ‘*a lot*’ over the past month^†^^1^. We also derived a second set of scores to capture ‘core’ *PTSD* symptoms that do not overlap with depression symptoms. For this, we recomputed our total symptom scores and binary variables excluding the five DAWBA PTSD items (7 items at 15 years) describing symptoms commonly also found in depression, such as reduced interest in activities, and trouble sleeping or concentrating, while retaining 8 items related to PTSD only, indexing, for example, intrusive re-experiencing or avoidance. This was done to check whether the well-established emergence of sex differences in depressive symptoms might be contributing to any PTSS effects as assessed via the DAWBA.

#### Age at peak-height velocity

Age at peak-height velocity (APHV) denotes the point of the maximum growth-spurt and has been proposed to be a more objective and non-invasive indicator of age of sexual maturation than the commonly used Tanner stages (Khairullah *et al*., 2013). In ALSPAC, APHV was derived using a superimposition by translation and rotation mixed effects growth curve analysis based on repeated height measurements through development (Frysz *et al*., 2018). The resultant measure was used to investigate the influence of pubertal status as a moderator of sex differences in PTSS^2^.

#### Additional variables

Information on the sex of the child was obtained from birth notifications, and demographic variables (household income, highest maternal educational qualification, child ethnicity, maternal relationship status and maternal smoking) were collected during pregnancy or shortly after delivery via maternal report. In order to examine the possibility of selective attrition, we used data from the Strength and Difficulties Questionnaire (SDQ) (Goodman, 1997) collected from parents at 8 years, as an extremely well-established index of child emotional and behavioural problems.

### Data analysis

Data were analysed using Stata 14 (Stata Corp., 2016). We compared participants included in analyses with those excluded due to missing data using χ^2^ and *t* tests as appropriate. Furthermore, we used logistic regression to test for sex differences in exposure to traumatic events. Levels of PTSD symptoms in males *v.* females were compared using Mann-Whitney-*U* tests, as they were not normally distributed. We tested for sex differences in overall PTSS and PTSD core symptoms in participants exposed to trauma at each age, using logistic regression, adding APHV as a moderator in a second step by including an interaction term.

## Results

### Descriptive statistics

**Fig. 1.**
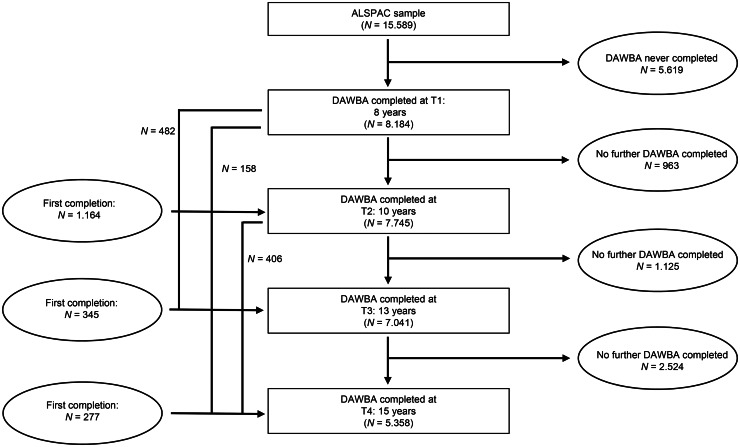
DAWBA PTSD-assessment availability for the ALSPAC cohort at ages 8, 10, 13 and 15, based on the *N* = 15.589 children for whom initial assessments were collected.

Figure 1 summarises the number of participants and observations available at each age; 9966 participants completed at least one DAWBA assessment across all 4 time-points, 50.7% males (*n* = 5051) and 49.3% females (*n* = 4915). DAWBA PTSD assessments at every age (8, 10, 13 and 15 years) were available for a core group of *n* = 3741 children and adolescents. For an additional *n* = 1756, all three parent ratings were completed (ages 8, 10 and 13), leading to a full parent-report sample of *n* = 5497. Further descriptive data can be found in Table 1.

**Table 1.** Sample descriptive data (*N* = 9966) based on assessments during pregnancy/shortly after birth

Demographic variable	% (*N*)
Family income GBP per week	
<100	7.5 (566)
100–399	67.4 (5105)
>400	25.1 (1904)
Highest maternal educational qualification	
GCSE	15.5 (1423)
O Level or A Level	60.4 (5543)
Vocational training	9.1 (840)
Higher education	15.0 (1377)
Mother primiparous	46.2 (4200)
Mother ever smoked	46.7 (4340)
Partner living in home at time of pregnancy	93.2 (8712)
Child ethnicity	
White	98.1 (8957)
Black	0.6 (58)
Chinese or South East Asian	0.7 (65)
Other	0.6 (55)

#### Attrition

Attrition from the ALPSAC sample has been found to be systematically related to factors such as larger family size, financial difficulties, and the mother being single, a smoker and not having educational qualifications (Howe *et al*., 2013). In addition, we found lower SDQ total problem scores as rated by parents at the age of 8 in those who completed at least one more DAWBA assessment after the age of 8 *v.* those who did not complete any further assessments (*M*_retained_ = 6.30, *M*_dropout_ = 7.24, *t*_(5828)_ = 3.09, *p* = 0.001). Similarly, those who had been exposed to a potentially traumatic event at 8 years were more likely to have no subsequent DAWBA assessments than those not exposed (14.6% of exposed children dropping out *v.* 11.4% of non-exposed, χ^2^ = 9.42, *p* = 0.002). However, no sex differences in the proportions of trauma exposed dropping out were found at any age (*p* > 0.21–0.81).

### Trauma exposure rates

Child trauma exposure rates by sex are presented in Table 2, based on parent report at 8, 10 and 13 years, and self-report at 15 years. Logistic regressions showed there were no differences in exposure rates between males and females at 8, 10 or 13 years (see Table 2), but at 15 years females were 35% more likely to report trauma exposure than males. The majority of events indexed were rated as ‘severely distressing’ (parent report 8 years: 69.2%, 10 years: 72.1%, 13 years: 70.1%; adolescent self-report 15 years: 78.6%).

**Table 2.** Trauma exposure rates, according to age and sex

		Trauma exposure [%, (N)]	Total sample *N*	OR	OR- 95% CI	χ^2^	*p*
8 years	Female	12.7 [506]	3976	1.02	0.90–1.17	0.12	0.73
	Male	12.5 [525]	4208				
10 years	Female	15.9 [610]	3838	1.00	0.89–1.13	0.00	0.99
	Male	15.9 [621]	3907				
13 years	Female	17.1 [598]	3506	1.09	0.96–1.24	1.80	0.18
	Male	15.9 [561]	3535				
15 years	Female	13.8 [387]	2817	1.35	1.15–1.60	12.87	<0.001
	Male	10.5 [267]	2536				

OR, Odds ratio; CI, Confidence interval.

*Notes*. The 15 years-assessment was obtained via self-report, while all other reports are parent-based.

### PTSS scores

For the subgroup of children and adolescents who had been exposed to trauma, current symptom scores at each time point are presented in Table 3. Average current symptoms were low at all time-points but spanned a wide range. No sex differences in symptom levels were found at 8 years, *Z* = −0.47, *N* = 1031, *p* = 0.642, or 10 years, *Z* = −0.10, *N* = 1231, *p* = 0.925. However, Mann-Whitney *U* tests revealed higher symptoms in females than males at 13 years, *Z* = −2.91, *N* = 1159, *p* = 0.004, and 15 years, *Z* = −3.98, *N* = 654, *p* < 0.001. When analyses were rerun excluding symptoms that show strong overlap with depression, the pattern of results remained the same, with females having higher core PTSD symptom scores at 13 years, *Z* = −4.14, *N* = 645, *p* < 0.001, and 15 years, *Z* = −4.12, *N* = 654, *p* < 0.001, but not at 8 years *Z* = −1.36, *N* = 1031, *p* = 0.17 or 10 years *Z* = −0.1.44, *N* = 1231, *p* = 0.15.

**Table 3.** DAWBA mean symptom scores and current presence of PTSD symptoms by age and sex, based on trauma exposed subgroup at each time point

		Symptom scores				
Age	Sex	Mean	s.d.	Range	Proportion with symptoms %, [*N*]	*N* Trauma exposed
8	Female	2.96	3.79	0–22	26.5 [134]	506
	Male	2.75	3.21	0–22	24.4 [128]	525
10	Female	3.04	4.04	0–25	23.3 [142]	610
	Male	2.89	3.70	0–24	21.7 [135]	621
13	Female	3.26	4.05	0–23	28.2 [169]	598
	Male	2.56	3.46	0–20	20.3 [114]	561
15	Female	3.14	6.09	0–26	21.7 [84]	387
	Male	1.32	3.69	0–25	11.9 [32]	267

*Notes*. The 15 years-assessment was obtained via self-report, while all other reports are parent- based.

### Presence/absence of PTSS

Table 3 also presents the proportion of trauma exposed participants who did and did not report PTSS split by sex. For those classed as PTSS present (based on at least one symptom being experienced ‘a lot’) symptom scores were as follows: 8 years Mdn = 6, interquartile range (IQR) = 5–10, 10 years Mdn = 8 (IQR = 5–11); 13 years Mdn = 7 (IQR = 5–10); and 15 years Mdn 12 (IQR = 8–16). Binary logistic regressions were performed, using sex (0 = *male*, 1 = *female*) as a predictor for category membership of ‘*PTSS present*’, (0 = *no*, 1 = *yes*). Results showed that sex was not associated with PTSS at 8 years (χ^2^ = 0.60, *p* = 0.44; OR 1.12, 95% CI 0.84–1.48) or 10 years (χ^2^ = 0.042, *p* = 0.5; OR 1.09, 95% CI 0.84–1.43), but at age 13, females were more likely to report PTSS (χ^2^ = 9.84, *p* = 0.002; OR 1.54, 95% CI 1.17–2.02). Similarly, at 15-years females were more likely to self-report PTSS than males (χ^2^ = 10.64, *p* = 0.001; OR 2.04, 95% CI 1.31–3.32). Furthermore, when we repeated analyses examining only core PTSS (i.e. removing overlapping depression items), the pattern was almost identical; there were no sex differences at 8 years (χ^2^ = 1.16, *p* = 0.28; OR 1.18, 95% CI 0.87–1.61) or 10 years (χ^2^ = 0.90, *p* = 0.34; OR 1.15, 95% CI 0.86–1.52), but females were more likely to be symptomatic at 13 years (χ^2^ = 8.53, *p* = 0.004; OR 1.53, 95% CI 1.14–2.04) and at 15 years (χ^2^ = 10.33, *p* < 0.001; OR 2.01, 95% CI 1.31–3.30).

### Moderation by pubertal status

Pubertal status as indicated by APHV was available for a subset of *N* = 5707 participants of the overall study sample (2685 males and 3018 females). APHV means were *M* = 13.53, s.d. = 0.86 (range: 10.84–16.56) for males and *M* = 11.08, s.d. = 0.82 (range: 9.01–14.58) for females. Moderation analyses were performed for ages 13 and 15 only, as these were the only ages at which sex differences in PTSS were found. Binary logistic regression found no evidence of an interaction between sex and APHV at 13 years (*B*_interaction_ = 0.05, s.e. = 0.21, *p* = 0.804) in predicting presence/absence of PTSS. However, there was evidence of an interaction between sex and APHV at 15 years (*N* = 588, χ^2^ = 13.68, *p* = 0.003; *B*_interaction_ = 0.58, s.e. = 0.29, *p* = 0.046). Follow-up regression analyses revealed APHV was negatively related to whether or not PTSS were present in males (*B* = −0.44, s.e. = 0.24, 95% CI −0.91 to 0.03), while the association was small and positive in females (*B* = 0.14, s.e. = 0.26, 95% CI −0.17 to 0.45), indicating that having reached the point of maximum growth spurt later was associated with lesser symptoms in males. However, as confidence intervals each crossed zero, these effects should be interpreted with caution.

## Discussion

In a large longitudinal birth cohort, we found reliable evidence that sex differences in PTSS emerge during adolescence. Specifically, males and females showed similar risk of PTSS following trauma at 8- and 10-year assessments. However, from the age of 13 years females more frequently presented with PTSS after trauma exposure and showed higher average symptom levels. Sex differences were not accounted for by overlap between PTSS and depressive symptoms. In addition, we found little evidence that pubertal status accounted for heightened vulnerability to PTSS in adolescent females *v.* males.

Similar to previous cross-sectional analyses (Trickey *et al*., 2012), we found that from 13 years onwards, trauma exposed females were more likely to be symptomatic and exhibited higher average PTSS levels when compared to their male counterparts. By 15 years of age, females compared to males in the current study were approximately twice as likely to develop PTSS following trauma exposure, which is comparable to the sex difference reported in adult samples. Sex differences in PTSS did not appear to be secondary to overlap between PTSS and depressive symptoms, with the emergence of sex differences during adolescence in the latter being well-established (Wade *et al*., 2002). That we were able to study the same sample repeatedly from childhood to adolescence, to examine levels of PTSS only in the subgroup exposed to trauma, and that we could rule out sex differences in selective attrition related to PTSSS, are strengths that make the current findings particularly reliable.

The source of sex differences in PTSS/PTSD, and particularly the reason for their emergence in adolescence, remains to be explained. We focused on comparing symptoms within the subgroup of the ALSPAC sample that reported exposure to trauma, and we only found evidence of higher exposure rates in females at 15 years. The fact that sex differences were apparent *within* our trauma exposed subgroup means that differences in exposure rates are not an obvious explanatory factor. However, we cannot rule out unmeasured underlying sex differences in quality or extent of exposure. It has been proposed that females may be particularly vulnerable to developing PTSS following interpersonal trauma, and may experience different types of trauma to males (e.g. higher rates of sexual abuse in females), or be exposed to higher accumulated trauma over their lifetime (Breslau *et al*., 1999; Walker *et al*., 2004), although in the adult field such effects seem to offer limited explanation of sex differences. In the current study it was not possible to meaningfully categorise the type or severity of the young person's trauma exposure. Moreover, the fact that overall rates of reported exposure dropped between 13 years (parent report) and 15 years (adolescent report) was unexpected and underscores the need for more comprehensive measurement of traumatic events. We strongly recommend that future studies include standard trauma checklists to examine possible sex differences in exposure comprehensively.

We examined pubertal status as a key, sex-related biological development, and found little evidence that pubertal development as assessed via APHV contributes to sex differences in PTSS at 13 years. At 15 years, a later pubertal onset appears to be protective against PTSS development in males. The equivalent effect in females was extremely small and positive in direction. Overall, these findings do not offer a clear explanation of the relatively increased risk of PTSS in females during adolescence. It is important to note that APHV may indicate an earlier stage of puberty for females *v.* males (Granados *et al*., 2015); in females most growth and development of sex characteristics occurs in early puberty, before menarche, whereas in boys substantial hormonal changes occur before physical signs are present. As such, APHV is likely to co-occur with Tanner stages 1–3 in girls – and to be more closely coupled with endocrine changes – *v.* stages Tanner stages 4–5 in boys. The complexity of assessing of puberty, as a multifaceted and gender specific process, means that the current findings must be interpreted cautiously. Nonetheless, we found no evidence to support the possibility that the onset of puberty particularly increases vulnerability to PTSS development in females. Measurement of hormones, such as estrogen and orexin, which have been implicated in symptom development, could yield different conclusions (Grafe and Bhatnagar, 2018).

Other factors may also explain sex differences in PTSS. Adolescent females may exhibit more problematic psychological responses to trauma than males. For example, adolescent girls may be particularly likely to cope with stress through rumination, which may prolong difficulties (Hampel and Petermann, 2005). More broadly, females may possess different biological vulnerabilities in terms of HPA axis, neural circuits and epigenetic factors (Olff *et al*., 2007; Garza and Jovanovic, 2017; Ramikie and Ressler, 2018). Socio-demographic variables, such as differences in the emotional support given to adolescent females *v.* males, may also all potentially drive sex differences (Green *et al*., 1991; Bernard-Bonnin *et al*., 2008). Nonetheless, at present sex differences in PTSS remain largely unexplained. The current study highlights adolescence as a key period during which sex differences emerge, and a potentially useful focus for future study of contributing factors.

The current study has several limitations. First, the ALSPAC sample is slightly more affluent than the UK population average, and dropout was higher amongst families experiencing more challenges, including those children who had previously been exposed to a traumatic event, meaning that findings may not generalise to higher risk groups. Second, the DAWBA was administered as a questionnaire, and without a checklist of traumatic events being provided, potentially leading to certain traumas meeting DSM criterion A not being reported, which would have resulted in no further exploration of PTSS. Similarly, there is a risk that events may have been reported that would not typically be classified as traumatic; previous research has found that a proportion of individuals report events that do not meet DSM 5 Criterion A in self-report PTSD measures (Norris and Riad, 1997). That said, quality checks conducted by the research teams indicated that most of the incidents reported can be considered potentially traumatic according to DSM criterion A. Third, interviews relied on parent informants for the first three assessment time points but on the adolescent's own report for the final time point. At age 15 (self-report) there appeared to be somewhat lower rates of both exposure to trauma and PTSS compared to 13 years, but this may be due to the change in informant across these time points. Of note, sex differences in self-reported PTSS at 13 (carer report) and at 15 years (self-report) were consistent, and therefore the emergence of sex differences in adolescence is not attributable to change in informant. Fourth, the APHV variable was only available for a subset of participants for whom a sufficient number of height measures had been collected. However, comprising over 3000 children and adolescents, our sample was still large enough to yield reliable results.

In sum, the current study used data from a large UK cohort to determine rates of trauma-exposure and PTSD symptom development, stratified by age and sex. We found that sex differences in PTSD appear to emerge between the ages of 10 and 13 years. However, the onset of puberty was not found to be a likely explanatory factor for increased female vulnerability in adolescence. Further investigation of the potential drivers of the emergence sex differences in PTSS is warranted. Characterizing such patterns may help to better understand the developmental origin of the sex gap in PTSD prevalence, and to derive adequate preventive measures and interventions.

## Footnotes

† The notes appear after the main text.

## Notes

1 Too few cases of probable PTSD were identified by the DAWBA to allow further analyses, which led to our focus on comparing groups with and without symptoms. Due to the highly skewed continuous symptom scores, we split the score as described. We also performed all analyses using a lower threshold, including all those who reported any symptoms over the past month (i.e., symptom scored 1 *v*. 2). Patterns of results found were identical to those reported here, supporting the validity of the results presented.

2 In the ALSPAC cohort, APHV correlates *r* = 0.79, *p* < 0.001 with age of menarche, and *r* = 0.60 with age of thelarche (females); and *r* = 0.56, *p* < 0.001 with age of pubarche for females, and *r* = 0.25, *p* < 0.001 for males. Age at menarche, thelarche and pubarche were derived based on multiple questionnaire responses as previously described (Lawn *et al.*, 2017).
